# The opioid epidemic and accessibility to free Wi-Fi: internet access is a human rights issue

**DOI:** 10.1186/s12954-024-01061-3

**Published:** 2024-08-21

**Authors:** Ehsan Jozaghi

**Affiliations:** Faculty of Dentistry, University of British Columbia, 2199 Wesbrook Mall, Vancouver, BC V6T 1Z3 Canada

## Abstract

The opioid epidemic has taken the lives of thousands of people across North America and Europe. Moreover, lack of housing, inflation, and a rapidly changing economy have affected millions of people, and many have become homeless. Many governments, researchers, health agencies, and not-for-profits have offered innovative ways to tackle this crisis, including many harm-reduction technologies that rely on Internet. In the age of the first artificial intelligence (AI) revolution, where reliance and accessibility to Internet have become a necessity for finding jobs, housing, affordable food, social services, social connection, and staying alive, the creation of free Wi-Fi zones around inner city neighborhood by towns and municipalities is not only a cost-effective way to reduce death, social costs, but a human rights issue during the initial stage of first A.I. revolution.

## Introduction

Many nations globally are experiencing an alarming overdose crisis linked to a highly toxic and unpredictable illegal drug market [1]. Vancouver’s Downtown Eastside of British Columbia, Canada, has reported an overdose death rate of 557 per 100,000 annually, while some nations in Europe, such as the Kingdom of Scotland, have reported 16.8 per 100,000 [1, 2].

Many governments have declared public health emergencies in response to the unprecedented number of overdose-related mortalities [3]. Since the public health emergency declaration, various levels of government have worked together on implementing numerous methods of containing this epidemic, including educating the public and creating awareness, increased monitoring of overdose-related events, overdose prevention sites, peer-based programs, and distribution of naloxone kits [4, 5]. Some jurisdictions have experimented with innovative“safe supply” initiatives [2].

However, despite the success of such harm reduction programs in reducing overdose deaths and connecting at-risk populations with appropriate services [6–15], using drugs alone due to stigma is very prevalent [16]. Subsequently, many people die of drug overdose [16]. Consequently, new technologies such as Brave and Lifeguard have offered innovative ways to tackle this issue where a drug user is connected to a supporter wherever and whenever they use drugs [16]. However, such technology relies on Wi-Fi, which many underserved and at-risk groups do not have adequate access to [16].

## Changing economy, A.I.‘s first revolution, and homelessness

Subsequent technological advancements over the past two decades and the evolution of Artificial intelligence (AI) technology increasingly highlight the growing need for fast internet access and Wi-Fi so jobs, social services, housing, and food can be adequately reached [17–19]. The AI First Revolution, similar to the First Industrial Revolution, has been linked to a shift to a knowledge-based economy that is changing the free-market economy, science, medicine, and society much faster than previous industrial revolutions [20, 21]. Moreover, the growing inflation, inadequate housing, and changing job market due to the initial stage of the first AI revolution may continue to affect many citizens [20–22]. Homelessness has risen in many industrial and advanced economies [22, 23]. The AI revolution has begun an irreversible process in which AI will take over many human tasks/jobs [20, 21]. Therefore, it is expected that without government support/interventions for more social programs (e.g., job training, social housing, and nutritional support) and basic income support, many people will become permanently homeless and suffer severe health outcomes [20, 23].

## Free Wi-Fi-zones and human rights

Therefore, there is a need for a free Wi-Fi-zone inner city neighborhood where many under-housed, poor, and at-risk citizens reside, assemble, and access social services more than ever. These Wi-Fi areas should include downtown areas in small and large municipalities, as homelessness has become prevalent across large and small cities and towns. The City of Vancouver is one such pioneering municipality that has recognized the need, and the elected city officials unanimously cast votes to examine the free Wi-Fi in public accessibility in the city’s poorest postal code [16]. This is particularly important during the initial stage of the first A.I. revolution, where many citizens will be affected by changing job market demands and increasing reliance on fast, reliable Wi-Fi to connect to the job market and access information [18, 19]. Unsurprisingly, Reglitz [24] has argued that internet and Wi-Fi access to those who cannot afford it is an essential service (e.g., food and water) “or at least of crucial importance, to enable the realization of human rights and the promotion of democracy. According to this human right, Internet access must be free in the sense of ‘unmonitored and uncensored’ and provided ‘publicly’ or ‘free of charge’ to those who cannot afford it” [24, p. 1].

## Conclusion

The General Assembly of the United Nations (UN) passed a resolution that was non-binding linked to “The Promotion, Protection, and Enjoyment of Human Rights on the Internet” [24, p. 1]. This crucial resolution, while initially intended to protect the existing offline rights online, coincidently contributed to the discussion around Wi-Fi accessibility as a human rights issue for those who cannot afford it [24]. Internet and Wi-Fi accessibility have become a life and death issue for many poor, under-housed, and at-risk citizens who rely on it to access social services, housing, food, jobs, and harm reduction services. Although the fluctuating possession of a mobile phone is well known among at-risk groups that often have it stolen, lost, or pawned/sold, the right to have free access to Wi-Fi services should not be linked to the eventual lack of a mobile phone. Therefore, small and large urban centers must follow Vancouver’s lead in initiating free zones to prevent premature death, reduce cost, and improve accessibility based on human rights principles adopted by the U.N. General Assembly.

## Data Availability

No datasets were generated or analysed during the current study.
